# Error associated with estimates of Minimum Infection Rate for Endemic West Nile Virus in areas of low mosquito trap density

**DOI:** 10.1038/s41598-019-55632-7

**Published:** 2019-12-13

**Authors:** S. Chakraborty, R. L. Smith

**Affiliations:** 1 Program in Ecology, Evolution & Conservation Biology, University of Illinois at Urbana-Champaign, Champaign, IL USA; 2 Department of Pathobiology, University of Illinois College of Veterinary Medicine, Urbana, IL USA

**Keywords:** Statistical methods, Viral infection

## Abstract

West Nile Virus (WNV) is a mosquito-borne infection that can cause serious illness in humans. Surveillance for WNV primarily focuses on a measure of infection prevalence in the *Culex* spp. mosquitos, its primary vectors, known as the Minimum Infection Rate (MIR). The calculation of MIR for a given area considers the number of mosquitos tested, but not the relative effort to collect mosquitos, leading to a potential underestimation of the uncertainty around the estimate. We performed Value of Information analysis on simulated data sets including a range of mosquito trap densities in two well-studied counties in Illinois between 2005 and 2016 to determine the relative error introduced into MIR associated with changing the density of mosquito traps. We found that low trap density increases the potential for error in MIR estimation, and that it does so synergistically with low true MIR values. We propose that these results could be used to better estimate uncertainty in WNV risk.

## Introduction

West Nile Virus (WNV) causes an infectious disease in birds, horses and humans that is transmitted by the bite of infected mosquitoes^1^. In 80% of human cases, the disease does not produce any symptoms, but in a subset of patients it can cause febrile illness, joint pain, fatigue and weakness and about 1 in 150 people develop encephalitis or meningitis^1^. The disease first emerged in the United States in 1999, following which it has spread across the contiguous states and is now considered established. The first report of WNV in the state of Illinois was among dead birds in 2001; it has since spread to almost all counties in the state. From 1999 until 2017, the CDC reports that Illinois has had 2,458 human cases of WNV, the 5^th^ highest number in the nation, including 1,553 cases of neuro-invasive disease^1^. It is a nationally notifiable disease and the state of Illinois has a surveillance system to monitor this pathogen, resulting in reports in 2017 of 90 human cases, 8 human deaths, 25 birds with WNV positivity and 2,022 positive mosquito batches^2^.

Birds serve as the main reservoir hosts for WNV. The virus, which belongs to the *Flaviviridae* family, is transmitted by mosquitoes in the genus *Culex*. Several species of *Culex* mosquitoes, such as *C*. *tarsalis*, *C*. *quinquefasciatus*, *C*. *stigmatosoma*, *C*. *thriambus*, *C*. *pipiens*, and *C*. *nigripalpus*, have been found to be able to transmit WNV^3^. Mosquitoes become infected when they feed on birds that harbor the virus; they are then capable of transmitting WNV to humans and horses^1^.

Surveillance for WNV is conducted primarily by testing mosquitoes, monitoring birds particularly in the family *Corvidae*, and sero-surveillance of sentinel chicken flocks and equines. The Illinois Department of Public health monitors WNV by testing groups of up to 50 mosquitoes, dead perching birds (such as crows, blue jays, and robins), and testing sick horses and humans with West Nile virus-like symptoms^2^. Since, individual collection and testing of mosquitoes can be an expensive and arduous process, pooled samples are used to detect the presence of pathogens within the species- a concept first introduced by Dorfman in 1943^4^. Mosquitoes are thus collected for testing by setting traps throughout the state; the placement of traps and number of mosquitoes submitted for testing is determined by local public health departments or mosquito abatement districts. The mosquito abundance in traps can be affected by factors such as temperature^5^, rainfall^6^, structure of urban landscapes^7,8^, vegetation^9^ and climatic variability^10^. Mosquitoes from these traps are collected in pools of up to 50 for viral testing. The results of mosquito testing are used to calculate the Minimum Infection Rate (MIR) for West Nile Virus, which is defined as the number of positive pools of a particular mosquito species over a defined time period and area divided by the total number of mosquitoes in those pools. The underlying assumption of MIR is that there is just one infected individual within a pool of mosquitoes. Another method used by researchers to detect the infection rate is using the Maximum Likelihood Estimate (MLE) which is defined as the infection rate most likely observed given the testing results and an assumed probabilistic model (i.e., binomial distribution of infected individuals in a positive pool)^11^. An increase in MIR estimates often is assumed to increase risk of disease transmission to humans. The Centers for Disease Control & Prevention have shown that MIR is an important indicator in WNV surveillance systems that can be helpful in predicting patterns in virus activity, and thereby human cases in a given area^12^. Previous studies that have used MIR to detect WNV are Bernard *et al*.^13^, Kulasekara *et al*.^14^ and Hadler *et al*.^15^. Therefore, prediction of MIR is a public health priority in areas endemic for WNV.

Under-sampling is believed to be a significant obstacle in developing robust prediction models for MIR. Under-sampling could be caused by not having enough traps set out within the geographic area of study, by not testing all mosquitoes collected in the traps, by low mosquito abundance in the traps, or by a combination of any of those factors. Additionally, MIR may not be the best measure of calculation if the virus to be detected is common or if pool sizes are too large^11^. Without sufficient data, MIR estimates are likely to be inaccurate and appropriate public health efforts will be difficult to determine. However, there is no current method for determining the error introduced into MIR by under-sampling.

Value of information analysis is a quantitative method to estimate the return on investment (value) produced by research^16^. This concept can be applied to compare the results of using an artificially reduced data set with that of the full data set to determine how much data are required to meet a specific criterion. The Value of Information (VOI) approach is increasingly becoming a useful tool with applications in prioritizing research decisions^17^, economic design of clinical trials^18^, as well as in treatment interventions^19^ and social sciences^20^. As the roots of VOI lie in statistics and has wide ranging applications, it is therefore used as a guiding concept in this paper to deal with error associated with MIR. In this case, the value we are interested in estimating is the accuracy and precision of the MIR estimate, while the information we are using is varying mosquito trapping densities.

The objective of this study was to determine the error associated with calculation of WNV MIR at the county level in cases of low mosquito trap density. This analysis considered data obtained from mosquito traps that were set in different locations in Illinois between the periods of 2005–2016. Utilizing the value of information concept as outlined above, we determined the impact of low trap density (under-sampling) in the accuracy of MIR results.

## Results

After selecting only weeks in which at least 50 pools of mosquitos were tested and at least one pool was positive, a total of 240 weeks of data were available for Cook County and 182 weeks of data were available for DuPage County (Table 1).

**Table 1:** Description of observed and simulated data (median [interquartile range (IQR)]) used for estimating baseline MIR and to determine effect of lower density sampling.

County	County		Proportion of Traps Sampled					
	Cook	DuPage	50	60	70	80	90	100
Number of Weeks	240	182	422	422	422	422	422	422
Number of Positive Pools per Week	21 [3, 103]	4 [1, 17]	5 [1, 23]	6 [1, 28]	7 [1, 33]	7 [1, 37]	8 [1, 42]	9 [1, 47]
Total Number of *Culex* Mosquitos per Week	12381 [7130, 18232]	2796 [2075, 3681]	2677 [1360, 6972]	3220.5 [1635, 8390]	3770 [1907, 9771]	4308 [2182, 11236]	4850 [2478, 12610]	5423 [2780, 14041]
MIR	0.0022 [0.0003, 0.0045]	0.0014 [0.0003, 0.0080]	0.0019 [0.0002, 0.0078]	0.0019 [0.0002, 0.0078]	0.0019 [0.0003, 0.0079]	0.0019 [0.0003, 0.0079]	0.0019 [0.0003, 0.0079]	0.002 [0.0003, 0.0078]
Number of Traps Used per Week	373 [229, 506]	92 [67, 116]	84 [46, 202]	101 [55, 242]	118 [64, 282]	134 [73, 322]	151 [82, 363]	168 [91, 402]
Density of Traps (traps/mi^2^)	0.39 [0.24, 0.54]	0.28 [0.20, 0.36]	0.16 [0.11, 0.23]	0.19 [0.13, 0.28]	0.22 [0.15, 0.33]	0.26 [0.17, 0.37]	0.29 [0.2, 0.42]	0.32 [0.22, 0.47]
Probability of False Negative	NA	NA	0.09	0.06	0.04	0.03	0.01	NA

The probability of a false negative was calculated as the number of iterations in which MIR was 0 when *MIR*_100_ was greater than 0.

**Figure 1:**
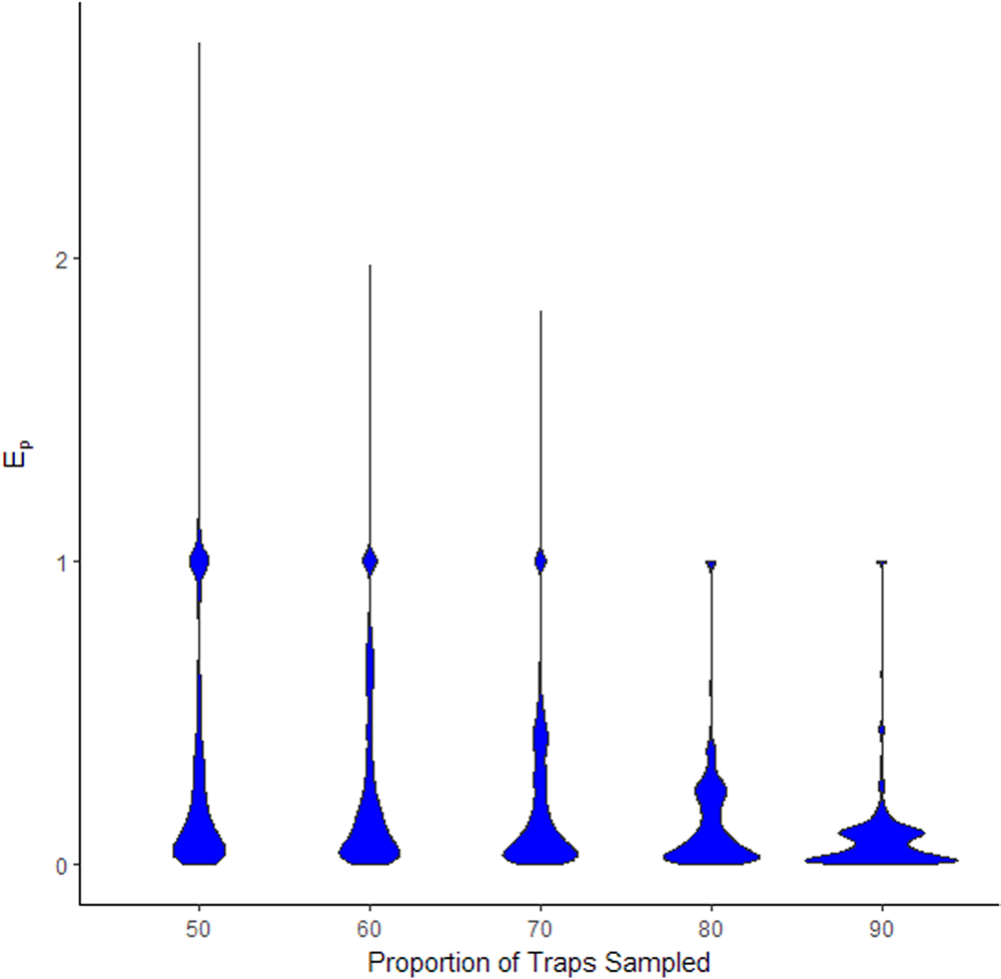
Distribution in simulated absolute relative error (${E}_{p}=|\frac{MI{R}_{p}-MI{R}_{100}}{MI{R}_{100}}|$) in MIR associated with sampling different proportions of mosquito trap data in Cook and DuPage counties on a weekly basis between 2005 and 2016. Each proportion of traps was randomly selected from all trap data available in a given week for 50 iterations.

**Figure 2:**
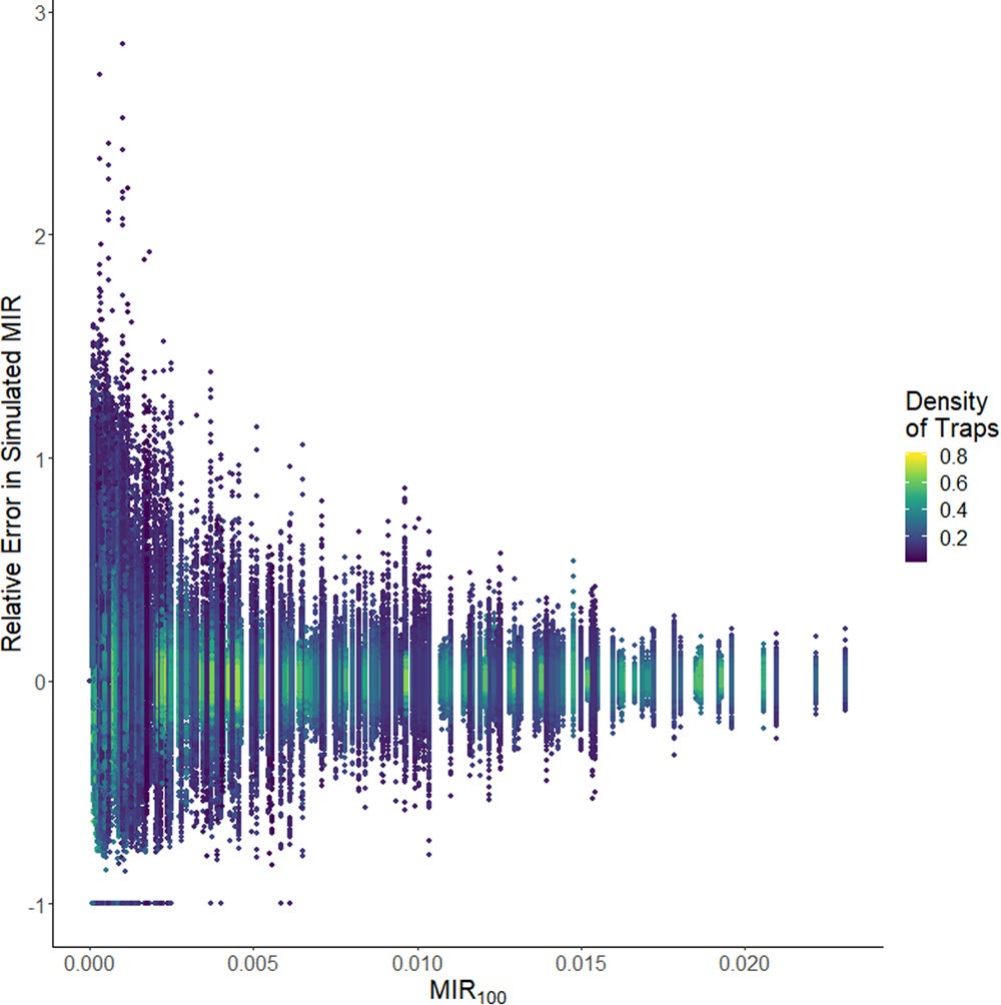
Simulated relative error in MIR for Cook and DuPage counties created by randomly sampling only a subset of mosquito trap data. Color indicates the number of traps per square mile in the simulated data. *MIR*_100_ is the observed MIR using all data.

After randomly sampling subsets of mosquito trap data, the absolute relative error in estimated MIR for a county, ${E}_{p}=|\frac{MIR-MI{R}_{100}}{MI{R}_{100}}|$, was clearly skewed, with a high concentration near 0 and a long tail (Fig. 1). There was a secondary peak in frequency near E_p_ = 1 due to the bounded distribution of MIR, which cannot fall below 0; any iteration in which no positive traps were sampled (probabilities shown in Table 1) would result in an E_p_ value of 1. This also resulted in a skew in the relative error, $\frac{MIR-MI{R}_{100}}{MI{R}_{100}}$ (Fig. 2) towards more positive outliers.

**Figure 3:**
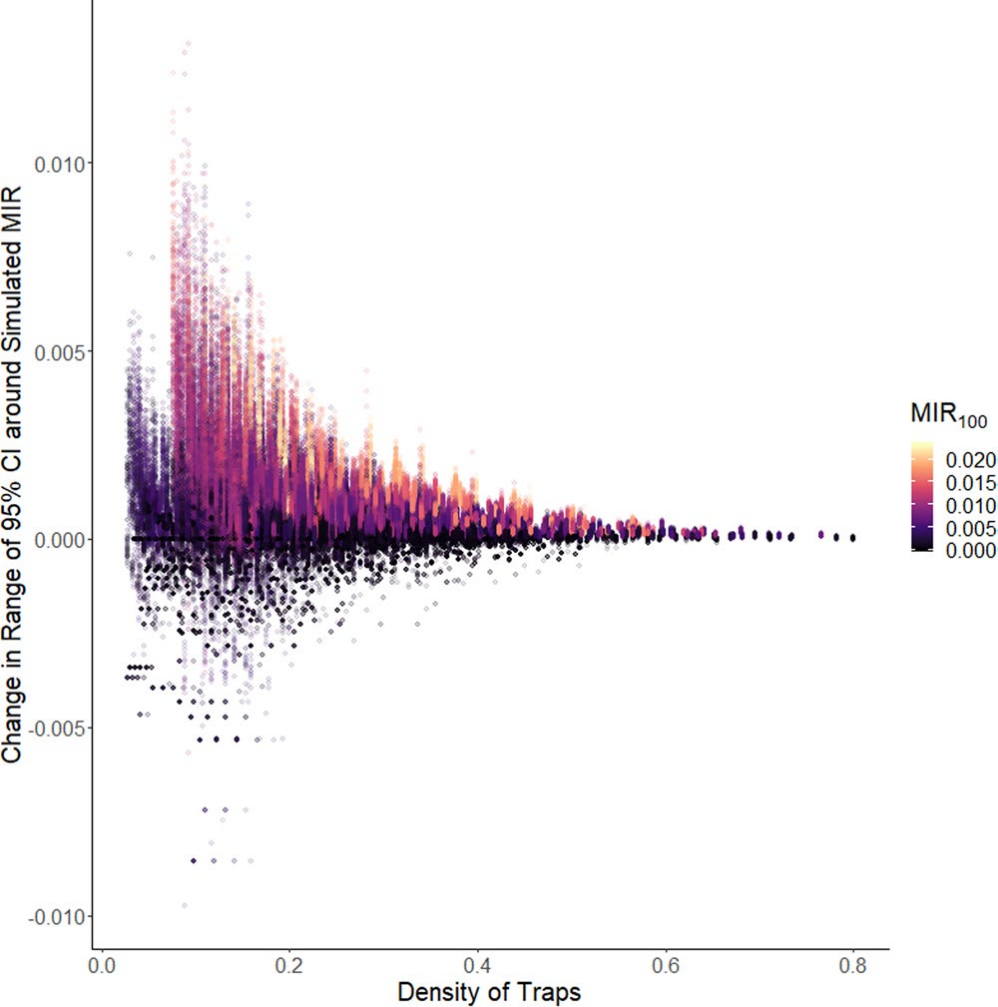
Change in the range of the 95% confidence interval around the simulated MIR for Cook and DuPage counties created by randomly sampling only a subset of mosquito trap data, as a function of the number of traps per square mile in the simulated data. Color represents *MIR*_100_, the observed MIR using all data.

The distribution of relative error in MIR was clearly wider when the density of traps decreased, and also when the observed *MIR*_100_ was low (Fig. 2). Results of a lognormal regression to determine the effect of trap density and *MIR*_100_ on relative absolute error showed that there was a significant synergy between the variables (Table 2). Likewise, the range of the 95% confidence interval around the MIR estimate tended to increase more when the density of the traps decreased (Fig. 3), but a higher estimate of *MIR*_100_ was associated with a higher range around the MIR estimate, although this effect was somewhat decreased with high trap density (Table 3). Information about model fit can be found in the Supplementary Information.

**Table 2:** Results of lognormal regression for absolute relative error in estimated MIR, $log\,(MIR+0.00001) \sim {\beta }_{0}+{\beta }_{1}MI{R}_{100}+{\beta }_{2}Density+{\beta }_{3}\,MI{R}_{100}\,\ast \,Density+{\varepsilon }_{i}$.

Variable	Coefficient (*β*)	95% CI
Intercept	−0.24	−0.34, −0.15
*MIR*_100_	−130	−150, −120
Trap Density	−5.9	−6, −5.7
*MIR*_100_*Trap Density	−1.0	−16, 14

**Table 3:** Results of linear regression for range of the 95% confidence interval around estimated MIR, $(Range-Rang{e}_{100}) \sim {\gamma }_{0}+{\gamma }_{1}MI{R}_{100}+{\gamma }_{2}Density+{\gamma }_{3}\,MI{R}_{100}\,\ast \,Density+{\varepsilon }_{i}$.

Variable	Coefficient (*γ*)	95% CI
Intercept	0.00043	0.00035, 0.0005
*MIR*_100_	0.29	0.28, 0.3
Trap Density	−0.0013	−0.0013, −0.0012
*MIR*_100_*Trap Density	−0.69	−0.7, −0.68

**Figure 4:**
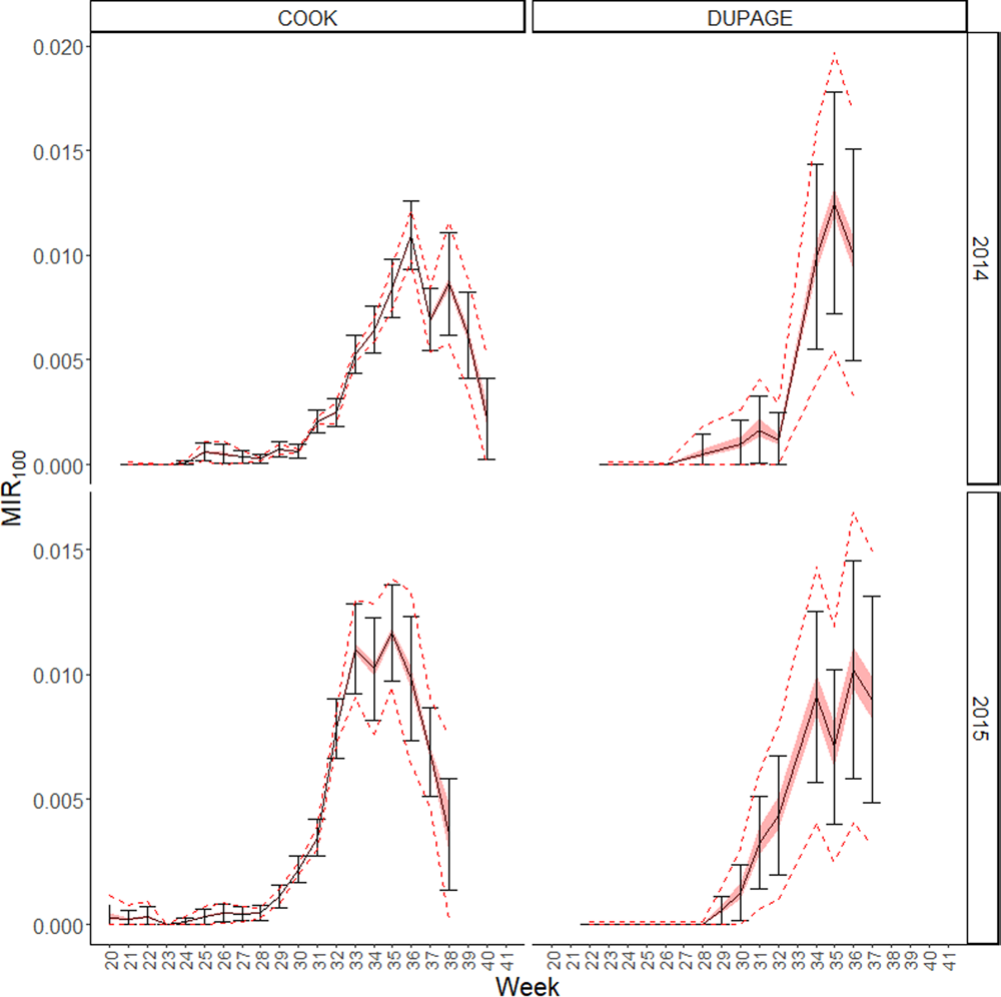
Predicted error associated with decreased West Nile Virus sampling in Cook and DuPage County for 2 example years. Black solid lines show the observed MIR with error bars showing the 95% confidence intervals, while the red shaded bar is the predicted error around the mean and the red dashed lines show the predicted 95% confidence intervals around the upper and lower bounds of the potential MIR estimate.

**Figure 5:**
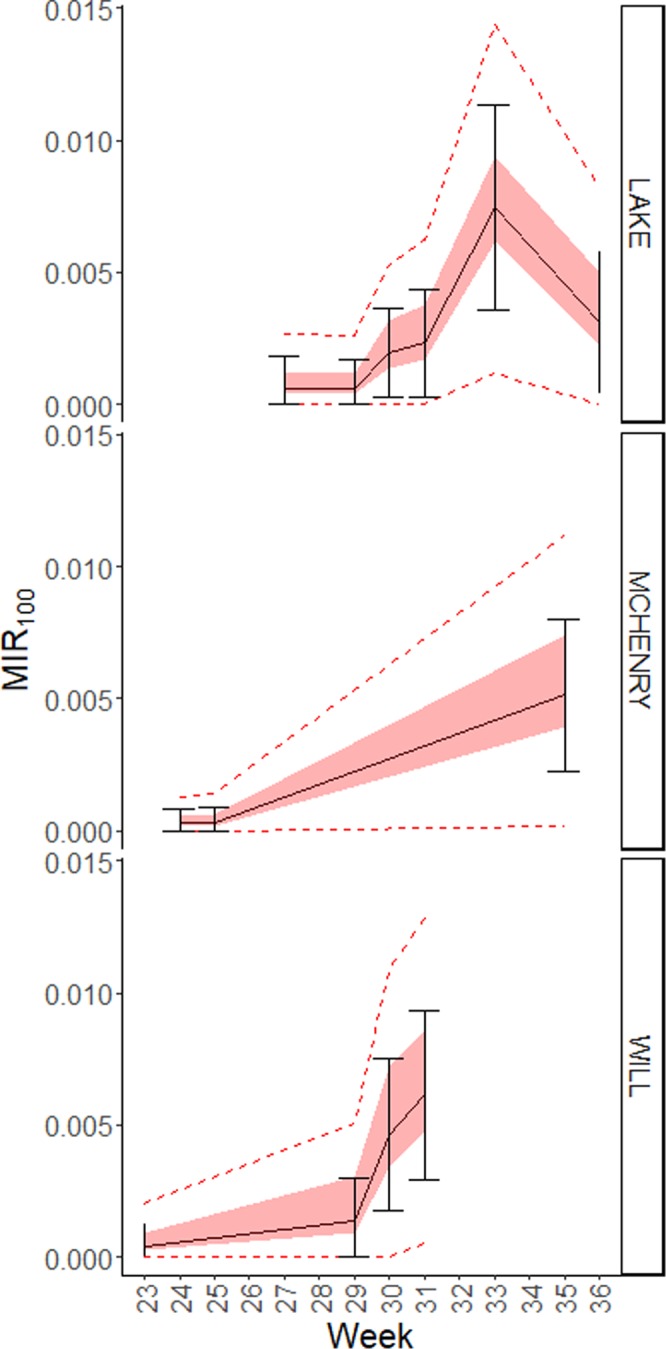
Predicted MIR error associated with trap density in Will, McHenry, and Lake Counties for one example year each. Black solid lines show the observed MIR with error bars showing the 95% confidence intervals, while the red shaded bar is the predicted error around the mean and the red dashed lines show the predicted 95% confidence intervals around the upper and lower bounds of the potential MIR estimate.

The equation from Table 2 could be used to calculate the expected potential absolute relative error (ARE) around an observed MIR by using the equation

$${E}_{p}=exp[{\beta }_{0}+{\beta }_{1}MIR+{\beta }_{2}Density+{\beta }_{3}\,MIR\,\ast \,Density]-0.00001$$

where ${E}_{p}=|\frac{MIR-MI{R}_{100}}{MI{R}_{100}}|$. The expected potential range of *MIR*_100_ can be calculated as $\frac{MIR}{1\pm {E}_{p}}$. Likewise, the expected change in the 95% confidence interval range can be calculated from the equation in Table 3. When the regression equation from Table 2 is applied to the observed data for Cook and DuPage counties, the range of predicted values is shown to be small(Fig. 4): at current trap density, the median range around the MIR is 0.18, which is only smaller than the observed 95% confidence interval, which has a median of 2.1. This is likely due to the high trap density, large number of pools, and high *MIR*_100_ in Cook County for this period. When the model is used to predict potential for error in example years from Will, McHenry, and Lake Counties(Fig. 5), where trap density is lower, it is seen that the error associated with MIR calculated from all traps is quite high; the median range around the MIR is 2.0. This is only slightly less than the observed 95% confidence interval, which has a median of 3.8, likely due to the small number of pools tested.

**Figure 6:**
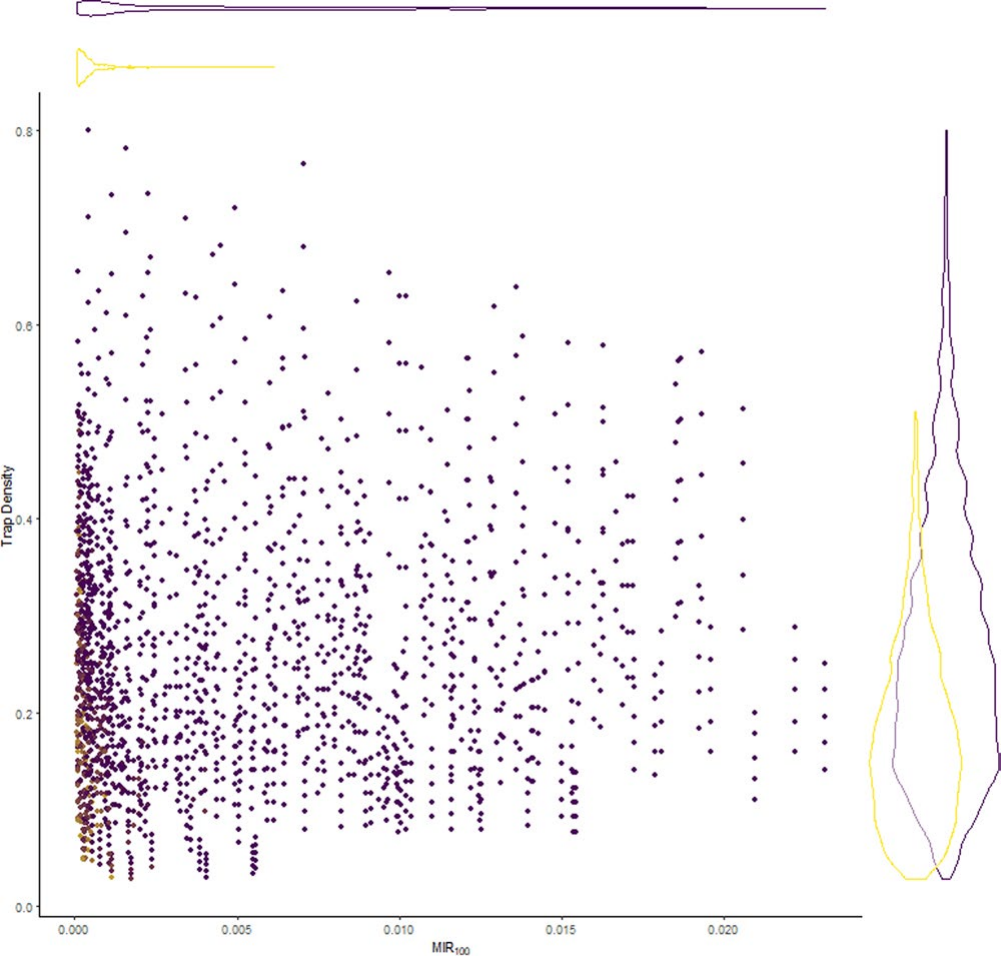
Simulated trap density in which the simulated MIR was 0 (in yellow) or non-zero (in blue) when the observed *MIR*_100_ was non-zero.

One factor of interest to mosquito control officials is the ability to detect a rise in MIR. We examined the simulated data for false negatives, circumstances in which the observed *MIR*_100_ was non-zero but the simulated *MIR*_*p*_ was zero (Fig. 6). As with the absolute relative error, the probability of a false negative was significantly increased with low trap density and with low *MIR*_100_, and the effect of trap density and *MIR*_100_ was synergistic (see Supplementary information). However, the effect of *MIR*_100_ was much greater than that of trap density.

## Discussion

There are various environmental factors that contribute to arbovirus transmission. Low levels of the virus might thrive within the host and reservoir populations and the exact conditions that lead to widespread outbreaks are difficult to pinpoint. Therefore, constant surveillance of mosquitoes and the sentinel organisms are a critical aspect of public health activities especially for West Nile Virus (WNV).

We found that if the true MIR is low, higher trap density is needed to accurately estimate MIR. This is intuitive; the sample size necessary to find a disease in a population is inversely proportional to the prevalence of the disease. Current estimates of MIR error are based on basic statistical theory, with sample size (total number of pools) responsible for much of the estimation^21^. This may result in underestimation of error around low MIR values and areas with low trap density but high trap numbers (such as a large geographical area).

Our study also provides an algorithm by which MIR error can be estimated. We believe that this error calculation can be incorporated into public health planning, giving decision makers a better sense of the potential range in MIR. Importantly, we have found that the probability of failing to detect a non-zero MIR was significantly impacted by the density of traps, especially when the actual MIR is low.

Our study was limited by the use of observed MIR, rather than true MIR, to calculate error. However, the mosquito surveillance systems of Cook and DuPage counties are extremely comprehensive, and the resulting calculated MIRs are likely to approach the true value in most cases. In fact, our model predicts low error in Cook County with only 10% of existing trap data, indicating that the full data set may be sufficient for the purposes of this study.

Researchers working on surveillance of arboviruses are constantly trying to optimize the tools and methods to better estimate disease risk and transmission in order to inform public health measures. Under-sampling has previously been identified as one of the most common sources of error in determining the mosquito infection rates^22^. For instance, DeFelice *et al*. (2017) used data assimilated from MIR and human case reports to model WNV transmission in New York and to generate retrospective forecasts of past WNV outbreaks in Long Island^23^. The authors realized for the model to work effectively in predicting outbreaks, it relied on timely availability of mosquito infection rate data, which can vary depending on the methods used for mosquito sampling. Bustamante and Lord (2010) also discussed other sources of error that are introduced while conducting mosquito surveillance such as temperature, trapping methods used for mosquito sampling, assays used for virus detection and the MIR vs MLE approach^24^. Thus, they suggest using other surveillance indicators such as historical baseline data and mosquito population size along with MIR for determining arboviral disease transmission.

The results of this study demonstrate that WNV surveillance in mosquitoes can be affected by under-sampling. However, the effect of this under-sampling on error in MIR estimates may now be calculated using our approach. It is important to note that we assumed that the spatial distribution of the traps was not a factor; this is a simplification that should be addressed in future studies. Gu *et al*. (2008) provide recommendations to better estimate MIR when there are not enough samples or in areas with low level transmission, recommending either “targeted surveillance (increased sampling at locations of higher transmission likelihood) or estimating MIR during periods of high transmission, thereby shifting from detection of mosquito infection to estimation of the transmission intensity, while expanding the number of sampling sites to evaluate the range of arboviral transmission”^25^. The placement of traps should also take into account landscape features and other factors likely to affect surveillance efforts.

Public health surveillance of diseases like West Nile virus and other mosquito-borne infections is essential and can be conducted smoothly via concerted efforts of public health agencies, health departments and research institutions. The setup of adequate number of traps in different counties to regularly monitor the MIR in the mosquito populations is critical so that necessary public health efforts can be initiated before outbreaks occur. This paper shows that in areas where trap density is low, the method utilized here can be used to detect accurately the error in MIR, which can inform appropriate disease control and prevention measures.

## Methods

Data were obtained from the Illinois Department of Public Health mosquito surveillance database, which collects mosquito trap testing information from major stakeholders such as public health departments and mosquito abatement districts in the state of Illinois. Mosquito trap data for 4 counties in Illinois (DuPage, Cook, Will, and Lake) were obtained for the years 2005 to 2016. Trap density (traps per square mile) was calculated for each week by dividing the number of traps tested by the total area of the county. All analyses were performed in R^26^. Analysis was performed using data from Cook and DuPage counties; all other county data were used for illustration of potential impact. Only weeks in which at least 50 pools were tested were included in the analysis.

For each county in each week, all data from *p* percent of traps (p ∈ {50%, 100%}) were removed at random and the remaining trap data were used to calculate the simulated *MIR*_*p*_ using the binGroup package^27^. Simulated *MIR*_*p*_ was compared to the observed MIR with 100% of data, *MIR*_100_, for that county-week combination, and absolute relative error was calculated as ${E}_{p}=|\frac{MI{R}_{p}-MI{R}_{100}}{MI{R}_{100}}|$. This was repeated 50 times for each county-week combination. Weeks in which *MIR*_100_ was 0 were removed from the analysis.

The error *E*_*p*_ was examined visually for each level of *p* and determined to be log-normally distributed with zero-inflation using the fitdistrplus package^28^. Due to the zero-inflation, we performed shifted logistic transformation by adding a conservative number (0.00001) to all *E*_*p*_ prior to log transformation. The impact of trap density and *MIR*_100_ on log(*E*_*p*_) was analyzed using mixed linear regression modeling with the lme4 package^29^, using the county-week combination as a random effect to account for repeated sampling. Two-way interactions were included, and all effects were considered significant at the α = 0.05 level. The 95% confidence interval around *MIR*_100_ and *MIR*_*p*_ were calculated using the binGroup package^27^, and the difference in the confidence interval range was calculated as *range*_100_*-range*_*p*_. The impact of trap density and *MIR*_100_ on the difference in the confidence interval range was analyzed using mixed linear regression modeling, as described above. All figures were created using the ggplot2 package^30^.

## Supplementary information

Supplementary Information

## Data Availability

All data and analysis scripts are available at https://github.com/rlsdvm/MIR_VOI.
