# Supplementary material for: Error associated with estimates of Minimum Infection Rate for Endemic West Nile Virus in areas of low mosquito trap density
Source: Sci Rep. 2019 Dec 13;9:19093. doi: 10.1038/s41598-019-55632-7 (PMC6911069; doi:10.1038/s41598-019-55632-7)
Supplement: Supplementary file 1 — Supplementary Information [file 41598_2019_55632_MOESM1_ESM.docx]

**Supplementary Information: Error associated with estimates of Minimum Infection Rate for Endemic West Nile Virus in areas of low mosquito trap density**

Chakraborty S^1^, Smith RL*^2^

^1^Program in Ecology, Evolution & Conservation Biology, University of Illinois at Urbana-Champaign, Champaign, IL USA

^2^Department of Pathobiology, University of Illinois College of Veterinary Medicine, Urbana, IL USA

^*^corresponding author: [rlsdvm@illinois.edu](mailto:rlsdvm@illinois.edu); 217-300-1428

**Model Fit**

The model with random effects, which is characterized by the equation$log\left( MIR+0.00001 \right)\sim\beta_{0}+\beta_{1}{MIR}_{100}+\beta_{2}Density+\beta_{3}{MIR}_{100}*Density+\varepsilon_{i}$, provides predictions that more closely track with the observed relative absolute error than the model involving only fixed effects (Figure S1)

Figure S1: Predicted and observed relative absolute error by county using models with only fixed effects or with random effects by county, week, and year. Dots indicate a single simulated value; color indicates the value of MIR_100_. Black lines show the diagonal which would indicate perfect agreement.


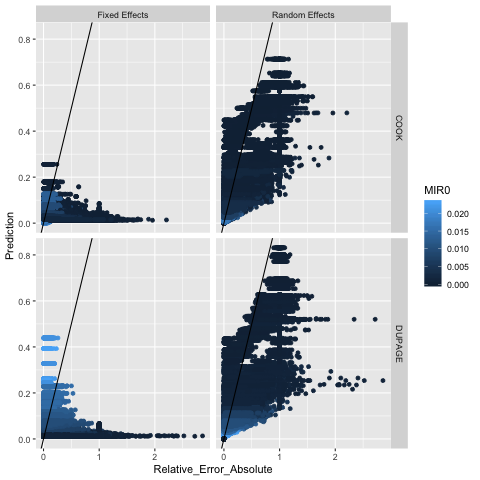


**Probability of False Negatives**

A logistic model with random effects, characterized by the equation

$logit\left( {MIR}_{p}=0|{MIR}_{100}>0 \right)\sim\beta_{0}+\beta_{1}{MIR}_{100}+\beta_{2}Density+\beta_{3}{MIR}_{100}*Density+\varepsilon_{i}$,

was fit to the simulated data described above to examine the effect of trap density and observed MIR on the probability of failing to detect a non-negative MIR. Results are shown in Table S1.

Table S1: Results of logistic regression for the probability of false negative MIR

| Variable | Coefficient | 95% CI |
| --- | --- | --- |
| (Intercept) | 0.11 | 0.04, 0.18 |
| MIR_100_ | -504 | -595, -416 |
| Trap Density | -4.27 | -4.6, -3.93 |
| MIR_100_*Trap Density | -10679 | -11398, -9960 |
